# Rhinosinusitis in hiv-infected children undergoing antiretroviral therapy

**DOI:** 10.1016/S1808-8694(15)30834-X

**Published:** 2015-10-18

**Authors:** Carlos Diógenes Pinheiro Neto, Raimar Weber, Bernardo Cunha Araújo-Filho, Ivan Dieb Miziara

**Affiliations:** 1 Physician and preceptor of the Clinical Otorhinolaryngology Division, Otorhinolaryngology and Ophthalmology Department, Faculdade de Medicina da Universidade de São Paulo (FMUSP).; 2 Graduate student, Clinical Otorhinolaryngology Division, Otorhinolaryngology and Ophthalmology Department, Faculdade de Medicina da Universidade de São Paulo (FMUSP).; 3 Graduate student, Clinical Otorhinolaryngology Division, Otorhinolaryngology and Ophthalmology Department, Faculdade de Medicina da Universidade de São Paulo (FMUSP).; 4 Livre-Docente certification professor at the Faculdade de Medicina da USP - Physician in charge of the ENT-HIV Group of the Clinical Otorhinolaryngology Division, Hospital das Clínicas da Faculdade de Medicina da USP. Technical and scientific director. Clinical Otorhinolaryngology Division, Otorhinolaryngology and Ophthalmology Department, Faculdade de Medicina da Universidade de São Paulo (FMUSP).

**Keywords:** children, haart, hiv infections, sinusitis

## Abstract

The association of protease inhibitors (PI) to antiretroviral therapy has generated sensible changes in morbidity and mortality of HIV-infected patients. **Aim:** Aims at evaluating the impact of this association on the prevalence of rhinosinusitis (RS) and CD4+ lymphocyte count in HIV-infected children. **Methods:** Retrospective cross-sectional study of the medical charts of 471 HIV-infected children. In 1996, protease inhibitors were approved for use as an association drug in antiretroviral therapy. Children were divided into two groups: one which did not receive PI and another which received PI after 1996. The prevalence of RS and CD4+ lymphocyte counts were compared between these groups. **Results:** 14.4% of HIV-infected children had RS. Chronic RS was more prevalent the its acute counterpart. Children under 6 years old who were taking protease inhibitors presented with a significant higher prevalence of acute RS. The association of PI with the antiretroviral regimen was associated to higher mean CD4+ lymphocyte count and lower prevalence of chronic RS. **Conclusions:** The use of protease inhibitors was associated to higher mean CD4+ lymphocyte count. Children under 6 years of age in antiretroviral therapy associated with PI presented a lower likelihood of developing chronic RS.

## INTRODUCTION

Worldwide dissemination of the Human Immunodefficiency Virus (HIV) has had a terrible impact on children. Based on 2004^1^ data, the UNAIDS/WHO has estimated that 2.2 million children aged below 15 years are infected. The Brazilian Ministry of Health reported 13 786 cases in children aged below 13 years by December 2004.^2^ The advent of new antiretroviral drugs, especially protease inhibitors, has substantially reduced the mortality and increased the life expectancy of these children.^3^, ^4^ This disease, together with the fact that many of the initial signs and symptoms of HIV infection occur in the ear, nose and throat, has meant that many such patients are brought in for assessment by otorhinolaryngolotists.^3^, ^5^, ^6^

Children generally present six to eight upper respiratory virus infections a year.^7^ About 5% to 13% of these episodes progress to secondary bacterial rhinosinusitis.^8^ Most of these children that develop a bacterial infection respond well to medication. Some, however, progress to chronic rhinosinusitis, particularly those with a dysfunction of nasosinusal physiology or any type of immune deficiency. HIV-infected patients are at an increased risk of developing chronic rhinosinusitis.^9^

Zidovudine, a nucleoside reverse transcriptase inhibitor (NRTI), was developed in 1987, and became the first medication for the Acquired Immunodefficiency Syndrome (AIDS). Non-nucleoside reverse transcriptase inhibitors (NNRTI) were developed next. In 1996, new antiretroviral drugs - the protease inhibitors (PI) - were introduced.^3^ The association of these new drugs (usually 2 NRTI + 1 PI or 1 NRTI + 1NNRTI + 1PI) is known as the Highly Active Antiretroviral Therapy (HAART).

Although rhinosinusitis is more prevalent in HIV-infected children, data on changes in the prevalence of this disease in children after HAART was introduced remain sparse.

Thus, the purpose of this study was to assess changes in the prevalence and pattern of rhinosinusitis and CD4+ lymphocyte serum counts in a specific population of Brazilian HIV-infected children following the introduction of HAART.

## MATERIAL AND METHOD

A cross-sectional study model was used to retrospectively review the files of 471 children aged from 0 to 12 years and 11 months with a prior diagnosis of HIV-infection seen at the HIV/AIDS Outpatient Unit from January 1990 to December 2004. Data analysis for this study was undertaken from January 1990 to December 2004; however, follow-up of these and new cases of HIV-infected children continues to done in our outpatient unit. The Research Ethics Committee of our institution approved the study (protocol number 153/03).

All children aged from 0 to 12 years and 11 months bearing a serological diagnosis of HIV infection (positive Elisa and/or Western Blot tests) that were seen at the outpatient unit from January 1990 to December 2004 were included in this study. Exclusion criteria were absence or irregular use of antiretroviral therapy or use of one or more antiretroviral drugs during 4 months or less. Twelve children were excluded from the sample. The 459 children included in the study were divided into two groups, as follows: those using regimens without protease inhibitors (Anti-Retroviral Therapy - ART) totaling 236 children, and those using HAART totaling 223 children. The mean duration of therapy among patients given ART was 14 ±4 months; among patients given HAART it was 9 ±2 months.

The same specialist (IDM) undertook a complete otorhinolaryngological physical examination in the first ambulatory visit. Rhinosinusitis was diagnosed and classified into acute and chronic forms according to the 1998 I Brazilian Consensus on Rhinosinusitis of the ABORL-CCF (I Consenso Brasileiro sobre Rhinosinusitis da ABORL-CCF). Thus, acute rhinosinusitis was diagnosed when children presented:^10^

a) A compatible medical history, duration up to 4 weeks (nose block, yellowish rhinorrhea, fever, headache, coughing and/or oropharyngeal irritation).
b) A suggestive physical examination (mucosal edema and hyperemia, purulent discharge in the nose, oropharyngeal secretion originating in the rhinopharynx and/or pain on palpating the area of the sinuses).
c) A plain radiograph of the paranasal sinuses in orthostatism (showing mucosal edema and/or sinus opacification).

Children were diagnosed as having chronic rhinosinusitis when presenting:^10^

a) A compatible medical history, duration over 4 weeks (yellowish rhinorrhea, coughing, cacosmia and/or feeling of heaviness in the head).
b) A suggestive physical examination (nasal and/ or retronasal purulent discharge).
c) Computed tomography of the paranasal sinuses (mucosal edema, opacification of sinuses and/or nasal cavity).

Some children underwent nasofibrolaryngoscopy, although this exam was not done routinely. A blood sample was taken to measure the serum CD4+ lymphocyte count on the same day of consultation.

Statistical analysis: Analysis of the 459 children was stratified by age, subdivided into two groups: 0 to 5 years and 11 months, and 6 to 12 years and 11 months. We calculated and compared the prevalence of rhinosinusitis in each age group, and the CD4+ T lymphocyte counts among ART and HAART patients. We applied the Pearson chi-square test and the Fisher exact test for categorical variables. For continuous variables we applied the Student t test for independent samples when there was homocedasticity and equality of variances; otherwise we used the non-parametric Mann-Whitney U test. P values below 0.05 were considered statistically significant. The software Statistical Package for Social Sciences (SPSS® for Windows 10.0, SPSS Inc., Chicago, IL) was used for data analysis.

## RESULTS

Table 1 shows demographic data (age, sex, origin and HIV infection route) and the antiretroviral regimens given to the 459 children. Immunological classification data about the children were taken from their medical files, and were based on the 1994 Center for Disease Control Revised Classification System for Human Immunodeficiency Infection in Children Less than 13 Years of Age, 11 these are summarized in Table 2. Among the 459 children, 66 (14.4%) presented rhinosinusitis, of which 24 (13.6%) were aged below 5 years and 11 months, and 42 (14.8%) were aged from 6 to 12 years and 11 months (p = 0.8). Thirty-six children (7.8%) presented chronic rhinosinusitis, and 30 children (6.5%) presented acute rhinosinusitis. Chronic rhinosinusitis was more prevalent in both age groups.

**Table 1:** Data on demography and antiretroviral regimens in 459 HIV-infected children.

Sex	

Male	258 (56.2%)
Female	201 (43.8%)

Age	

Mean ± Standard deviation (years)	6.6 ± 2.5
0 to 5 years and 11 months	176 (38.3%)
6 to 12 years and 11 months	283 (61.7%)

HIV - transmission	

Vertical	387 (84.3%)
Blood transfusion	18 (3.9%)
Indeterminate	54 (11.7%)

Origin	

Referred from Hospitals	362 (79.0%)
Household	97 (21.0%)

Antiretroviral therapy	

2 NRTI PI	133 (29.0%)
NRTI NNRTI PI	90 (19.6%)
AZT DDI	45 (9.8%)
AZT 3TC	82 (17.9%)
AZT only	90 (19.6%)
DDI only	19 (4.2%)

NRTI = Nucleoside Reverse Transcriptase Inhibitor: Zidovudine (AZT), Didanosine (DDI), Lamivudine (3TC); PI = Protease Inhibitor: Indinavir, Ritonavir, Saquinavir, Nelfinavir; NNRTI = Non-Nucleoside Reverse Transcriptase Inhibitor: Nevirapine, Efavirenz.

**Table 2:** Distribution of the 459-children sample into immune classes according to the age group and use or not of HAART.

Age group				
	0 to 5 years and 11 months (n = 176)		6 to 12 years and 11 months (n = 283)	
Immune class	HAART (n = 70)	ART (n = 106)	HAART (n = 153)	ART (n = 130)
A1	12 (17,1%)	5 (4,7%)	54 (35,3%)	22 (16,9%)
A2	32 (45,7%)	21 (19,8%)	28 (18,3%)	13 (10,0%)
A3	1 (1,4%)	-	-	-
B1	1 (1,4%)	-	36 (23,5%)	24 (18,5%)
B2	19 (27,1%)	42 (39,6%)	24 (15,7%)	37 (28,5%)
B3	1 (1,4%)	3 (2,8%)	-	1 (0,8%)
C1	-	-	1 (0,7%)	-
C2	3 (4,3%)	26 (24,5%)	7 (4,5%)	26 (20,0%)
C3	1 (1,4%)	9 (8,5%)	3 (2,0%)	7 (5,4%)

Table 3 shows the prevalences of rhinosinusitis and the distribution according to age and use of HAART. Among children aged from 0 to 5 years and 11 months, those that were given HAART had a higher prevalence of acute rhinosinusitis (12.9% vs. 1.9%, p=0.007). The relative risk of HAART-treated children aged below 5 years and 11 months of presenting acute rhinosinusitis was 2.2 times (95% CI: 1.6 - 3.1) the risk of ART-treated children presenting the same condition. The observed differences in the prevalence of chronic rhinosinusitis were not statistically significant.

**Table 3:** Prevalences of rhinosinusitis in the sample children according to age and use or not of HAART.

Age group						
	0 to 5 years and 11 months			6 to 12 years and 11 months		
	HAART (n=70)	ART (n = 106)	p	HAART (n = 153)	ART (n = 130)	p
Acute rhinosinusitis	9 (12.9%)	2 (1.9%)	0.007	11 (7.2%)	8 (6.2%)	0.9
Chronic rhinosinusitis	3 (4.3%)	10 (9.4%)	0.3	13 (8.5%)	10 (7.7%)	1.0

Total	12 (17.1%)	12 (11.3%)	0.4	24 (15.7%)	18 (13.8%)	0.8

HAART = *Highly Active Anti-Retroviral Therapy*

There were no statistically significant differences on the prevalences of acute and chronic rhinosinusitis among children given HAART and children given ART in the 6 to 12 years and 11 months age group.

Tables 4 and 5 present the mean serum CD4+ T lymphocyte counts in each age group. In the 0 to 5 years and 11 months age group children with chronic rhinosinusitis had serum CD4+ T lymphocyte counts on average 65.4 ±58 x 10-9cell/L below the count of children without rhinosinusitis (p = 0.002). There were no statistically significant differences among the mean serum CD4+ T lymphocyte counts among patients with or without acute rhinosinusitis in this age group.

**Table 4:** Serum CD4+ T lymphocyte counts according to the presence of rhinosinusitis in children aged from 0 to 5 years and 11 months.

	0 to 5 years and 11 months (n = 176)	
Rhinosinusitis	CD4 (x10-9 cell/L)	p
Acute		
Yes (n = 11)	846,9 ± 174,6	0,3
No (n = 165)	781,5 ± 189,5	
Chronic		
Yes (n = 13)	633,8 ± 187,7	0,002
No (n = 163)	797,7 ± 184,1

**Table 5:** Serum CD4+ T lymphocyte counts according to the presence of rhinosinusitis in children aged from 6 to 12 years and 11 months.

	6 to 12 years and 11 months (n = 283)	
Rhinosinusitis	CD4 (x10-9 cell/L)	P
Acute		
Yes (n = 19)	665,3 ± 131,1	<0,001
No (n = 264)	492,0 ± 183,1	
Chronic		
Yes (n = 23)	362,0 ± 136,4	<0,001
No (n = 260)	516,2 ± 183,8	

In children aged from 6 to 12 years and 11 months, those with acute rhinosinusitis had serum CD4+ T lymphocyte counts on average 173.3 ±42 x 10-9cell/L above the count of similarly aged children without acute rhinosinusitis (p < 0.001); children with chronic rhinosinusitis had CD4+ T lymphocyte counts on average 154.2 ±39 x 10-9cell/L below the count of similarly aged children without chronic rhinosinusitis.

Children given HAART had mean counts above the counts of children given ART, regardless of the age group; these differences were statistically significant (p < 0.001) and are shown on Table 6.

**Table 6:** Serum CD4+ T lymphocyte counts according to the age group and use of HAART.

Age group				
	0 to 5 years and 11 months (n = 176)		6 a 12 anos e 11 meses (n = 283)	
	CD4 (x 10-9 cell/L)	p	CD4 (x 10-9 cel/L)	p
HAART (n = 223)	872,7 ± 158,1	<0,001	539,1 ± 166,8	<0,001
ART (n = 236)	728,1 ± 186,0		461,9 ± 197,1	

HAART = Highly Active A

## DISCUSSION

Chronic rhinosinusitis tem has been reported as a frequent disease in HIV-infected adult patients.^4^, ^5^ Its prevalence, however, varies according to the study, probably due to different diagnostic methods for rhinosinusitis and different methods.^4^, ^7^ There are few published papers on the prevalence of rhinosinusitis in HIV-infected children; in these, results vary widely. Mofenson et al. found a 10.6% prevalence in 376 HIV-positive children. Chen et al. found a 20% prevalence in his series.^12^ Chaloryoo et al. studied 250 Thai children with AIDS and found a 0.8% prevalence.^13^ The prevalence of chronic rhinosinusitis among the 459 HIV-infected children referred to otorhinolaryngological evaluation in our study was 7.8%. Chaloryoo’s low prevalence stands out among these numbers. This may be explained by the fact that his series consisted of children with full-blown AIDS, therefore with more significant medical intercurrences such as hepatosplenomegaly (82.85%) and persistent pneumonia (64.4%). In this situation chronic rhinosinusitis may have been underdiagnosed.

It should be said that all patients in our study were being given antiretroviral therapy, albeit in heterogeneous regimens. In 1991, the Brazilian government started distributing Zidovudine free of charge to HIV-infected patients; the Law 9313/96 establishes that 100% of HIV-infected patients are assured free antiretroviral therapy, including protease inhibitors.

The prevalence in our series was higher in older children, which would be expected since the paranasal sinuses in younger children are not always fully developed.

There are no published studies comparing the prevalence of rhinosinusitis according to the type of antiretroviral therapy (HAART or ART). In our sample, children aged below 5 years and 11 months and given HAART had a higher prevalence of acute rhinosinusitis, thus a comparatively lower tendency to chronify. This may be due to the restoration of immune function in these children, who therefore respond more rapidly to germs in the paranasal cavities. Mizziara et al. studied the prevalence of otitis and found similar results: the prevalence of acute otitis media in the same age group was higher in children given HAART.^14^

The lack of any statistically significant difference in the prevalence of acute and chronic rhinosinusitis among children aged over 6 years, given HAART or ART, does not in itself suggest failure of highly active therapy (or even non-compliance to therapy); children who tended to chronify had lower mean serum CD4+ T lymphocyte counts.

Children with chronic rhinosinusitis in both age groups had lower serum CD4+ T lymphocyte counts compared to those without chronic rhinosinusitis. Castillo et al. studied adult patients with chronic rhinosinusitis and concluded that relapses were more frequent in patients with serum CD4+ T lymphocyte counts below 200/mL.^15^ In Barcelona, García-Rodríguez et al. studied 74 adult HIV-positive patients and found that low serum CD4+ T lymphocyte counts were associated with rhinosinusitis.^16^ Mofenson et al. studied 376 HIV-infected children and found that serum CD4+ T lymphocyte counts were not different in children with and without rhinosinusitis.^17^ In this study, the authors did not separate children with acute or chronic rhinosinusitis. The sample consisted mainly of younger children with acute rhinosinusitis. Mofenson et al.’s results are thus similar to ours in the group aged below 5 years and 11 months with acute rhinosinusitis; in this subgroup, serum CD4+ T lymphocyte counts were not different among children with and without rhinosinusitis (Table 4).

In our series the use of HAART improved the immune system of these children; their mean serum CD4+ T lymphocyte counts were higher compared to children given ART (Table 6).

Many factors may be involved for a nasosinusal condition to become chronic, such as the presence or absence of allergies or anatomical features that hinder the success of standard medical therapy.^18^

## FINAL COMMENTS

Our results show that rhinosinusitis in children aged below 5 years tended to chronify less often, probably due to an improved immune system. It should be noted, however, that given the cross-sectional nature of our study, these results do not imply causality. The number of children with rhinosinusitis was not that high, which may explain the lack of statistical significance in older children. Additional prospective studies are needed to assess more effectively the effect of HAART on the onset of rhinosinusitis in children.

In general these objections do not invalidate our study; it is, undoubtedly, one of the largest series of rhinosinusitis in HIV-infected Brazilian children.
